# Comment on “A Preclinical Systematic Review of Ginsenoside-Rg1 in Experimental Parkinson's Disease”

**DOI:** 10.1155/2017/7623954

**Published:** 2017-11-01

**Authors:** Yi-bo He, Yong-lin Liu, Yi-min Chen

**Affiliations:** Department of Clinical Lab, Zhejiang Provincial Hospital of TCM, 54 Youdian Road, Hangzhou 310006, China

We read the recently published systematic review of ginsenoside-Rg1 in experimental Parkinson's disease with a great deal of interest [1]. The authors concluded that G-Rg1 exerted potential neuroprotective functions against PD. However, the conclusion should be more conservative because the selection criteria in the meta-analysis are flawed and most preclinical studies of G-Rg1 in experimental Parkinson's disease have bias, which would decrease the reliability of these results. First, the authors chose TH-positive dopamine neurons and levels of TH protein in the SNpc as outcomes. However, loss of TH expression is not necessarily related to cells dying [2, 3], following MPTP and 6-OHDA. A temporal association of tyrosine nitration or cysteine oxidation with inactivation of TH activity in vitro suggests that this covalent posttranslational modification is responsible for the in vivo loss of TH function [4, 5]. So use of TH alone is insufficient to judge dopamine neurons loss; more outcomes should be added in this meta-analysis, such as numbers of Nissl stain-positive cells. Second, in Table 1, the authors did not state the timing of G-Rg1 treatment. Treatment with G-Rg1 before or after MPTP injection is totally different. We also reviewed included papers in this meta-analysis and found almost all studies pretreated with G-Rg1 before MPTP injection. It seemed that these studies did not strictly follow the protocol of Jackson-Lewis and Przedborski (2007) for the MPTP mouse model of Parkinson's disease [6]. All of them did not prove whether G-Rg1 would interfere with MPTP toxicokinetic or pretreatment, or whether coadministration with G-Rg1 may invalidate the interpretation of the data. It is uncertain whether G-Rg1 could prevent the uptake by blocking data, prevent the conversion of MPTP to MPP, detoxify MPTP, and many other possibilities. So, the method of pretreatment with G-Rg1 may not be scientific. Third, all studies in this meta-analysis count cell numbers immediately after the last injection of MPTP. This may lead to higher results as it takes time for cells to die and for the debris to be removed [2, 3], which could be an experimental flaw.

In conclusion, the authors have set out to prove the benefits of the G-Rg1 without critically reviewing the studies. The conclusion should be more conservative. Further, carefully controlled studies in animals should be attempted to see if G-Rg1 is a drug candidate rather than be confirmed by clinical trials immediately.
